# Cytokines, Chemokines, and Other Biomarkers of Response for Checkpoint Inhibitor Therapy in Skin Cancer

**DOI:** 10.3389/fmed.2018.00351

**Published:** 2018-12-12

**Authors:** Jennifer A. Bridge, James C. Lee, Adil Daud, James W. Wells, Jeffrey A. Bluestone

**Affiliations:** ^1^Diabetes Center, University of California, San Francisco, San Francisco, CA, United States; ^2^Helen Diller Family Comprehensive Cancer Center, UCSF, San Francisco, CA, United States; ^3^The Faculty of Medicine, The University of Queensland Diamantina Institute, The University of Queensland, Translational Research Institute, Brisbane, QLD, Australia; ^4^Sean N. Parker Autoimmune Research Laboratory, Diabetes Center, University of California, San Francisco, San Francisco, CA, United States

**Keywords:** biomarkers, checkpoint inhibitors, cytokines, chemokines, melanoma, Squamous cell carcinoma, Basal cell carcinoma, Merkel cell carcinoma

## Abstract

Immunotherapy for skin malignancies has ushered in a new era for cancer treatments by demonstrating unprecedented durable responses in the setting of metastatic Melanoma. Consequently, checkpoint inhibitors are now the first-line treatment of metastatic melanoma and widely used as adjuvant therapy for stage III disease. With the observation that higher tumor mutational burden correlates with a better response, checkpoint inhibitors are tested in other skin cancer types of known high tumor mutational burden with promising results and recently became the first-ever FDA-approved treatment for metastatic Merkel cell carcinoma. The emerging new standards-of-care will necessitate more precise biomarkers and predictors for treatment response and immune-related adverse events. Measurable immune-related mediators are currently under investigation as factors that promote or block the response to cancer immunotherapy and may provide insights into the underlying immune response to the tumor. Cytokines and chemokines are such mediators and are crucial for facilitating the recruitment and activation of specific subsets of leukocytes within the microenvironment of skin cancers. The exact mechanisms of how these meditators, both immunological and non-immunological, operate in the tumor microenvironment is an area of active research, so to reliable biomarkers of responses to cancer immunotherapy. Here, we will review and summarize the expanding body of literature for immune-related biomarkers pertaining to Melanoma, Basal cell carcinoma, Squamous cell carcinoma, and Merkel cell carcinoma, highlighting clinically relevant checkpoint inhibitor therapy biomarker advancements.

## Introduction

In recent years, the field of cutaneous oncology has been invigorated by novel therapies that modulate the immune system, and shifted away from conventional chemotherapy, radiation, and targeted therapy. Immune checkpoint inhibitors (CPIs) are at the center of this new treatment paradigm, providing unprecedented rates of response in Malignant Melanoma (MM), Basal cell carcinoma (BCC), Squamous cell carcinoma (SCC) and metastatic Markel cell carcinoma (mMCC). The most impressive clinical difference of immune-based therapies in comparison with targeted therapy is the durability, while the majority of the complete responder treated with CPIs still cancer free (>60%) (1) vs. <30% at 4 years treated with dabrafenib plus trametinib, a regimen for BRAF-mutated patients (2). However, despite the promising results of CPI treatment in several cancers, only a subset of patients show long-term response, and CPI-based therapies can result in severe and potentially life-threating side effects termed immune-related adverse events (irAEs). The overall incidence rate of irAEs has been reported occur in up to 90% of patients treated with anti-CTLA-4 (3) and 70% of patients treated with PD-1 or PD-L1 antibodies (4, 5), with serious toxicities (> grade 3) needing treatment discontinuation for anti-PD-1 antibody therapy, anti-CTLA-4 antibody therapy, and combination therapy at 21, 28, and 59%, respectively, in large phase III trial analyses(6, 7). It is still unclear of the mechanisms of irAEs and why some patients develop them, nor why certain CPIs cause certain irAEs or their effect on patient outcomes. Hence, predictive biomarkers that can aid in the more precise delivery of immunotherapy to patients are urgently needed. Thus, far, few biomarkers have been established that can predict treatment success and positive clinical outcomes for patients. Mutations in tumor cells are thought to be a mechanistically relevant and an important initial event for the generation of neoantigens that contribute to the initial anti-tumor immune response (8–12). Tumor mutational burden (TMB) became one of the first analytic tools to have a significant correlation with response rate and prognostication (10, 11, 13). Programmed death-ligand 1(PD-L1) is a transmembrane protein, capable of being expressed by most tissues, that plays a major role in suppressing the immune system via engagement of programmed death protein 1 (PD-1) expressed on activated T cells. The inflammatory cytokines produced following T cell activation results in the expression of PD-L1 in the surrounding tissue and promote selective immune tolerance. In the tumor microenvironment, PD-L1 can be overexpressed by the tumor cells and antigen-presenting cells (APCs), in turn blocking the appropriate T cell immune responses required for tumor rejection. PD-L1 expression is currently the only U.S. FDA approved, commercially available predictive tool that helps identify patients who are likely to benefit from therapy that blocks the programmed death protein 1 (PD-1)/PD-L1 axis pathway (14). Both biomarkers serve to estimate the statistical likelihood of treatment success, but seldom are either used in practice as the major clinical decision factor for which to deploy CPIs for a patient, perhaps due to their relatively low positive predictive value and undefined or sometimes controversial correlation with overall survival (15, 16). As more sophisticated means of patient immune monitoring, both on and off therapy, are being developed, other potential biomarkers are being investigated. In addition, it is worth noting that although a number of reviews have addressed the importance of chemokines and cytokines in skin cancer progression and metastasis (17–23), fewer, if any, have addressed their potential as biomarkers for treatment outcomes in skin malignances. Here, we offer a brief overview of the current and emerging standard-of-care drugs used in cancer immunotherapy for metastatic skin cancers, and provide updates on immune-related biomarker advancements pertaining to MM, BCC, SCC, and mMCC, including TMB, PD-L1, novel immune activation/exhaustion markers, as well as relevant cytokines/chemokines, highlighting research that may be invaluable for the diagnostic, prognostic and predictive information helpful in establishing better clinical outcome.

## Mechanism of Tumor Immune Escape and Clinical Impact of Immune Checkpoints in Metastatic Skin Cancer

CTLA-4 and PD-1 are both elements of a naturally evolved network of peripheral tolerance responsible for maintaining immune homeostasis and preventing overt autoimmunity. Early studies in animal models showed that cancers exploit these regulatory mechanisms, as checkpoint proteins are frequently over expressed in the tumor microenvironment. CTLA-4 is upregulated during T cell activation and eventually outcompetes costimulatory molecule CD28 for CD86 and CD80 expressed on antigen-presenting cells, halting further activation (24–26). CD86 and CD80 are known to be up-regulated at sites of inflammation and are capable of being removed from the cell surface through process known as trans-endocytosis by CTLA-4-expressing cells as a method also of block CD28 co-stimulation (27). In addition, CTLA-4 engagement enhances T_reg_ suppressive function (28). Anti-CTLA-4 antibodies are capable of blocking and preventing this interaction, effectively shifting the balance back toward T cell activation (3, 4, 29). Pre-clinical and clinical data show CTLA-4 blockade results in the activation of both CD4 and CD8 effector cells in favor of anti-tumor immunity. The effect on the suppressive capacity of regulatory T cells in the tumor microenvironment remains controversial and is an area of active investigation (30–32). While the CTLA-4 pathway appears to regulate anti-tumor immunity in the draining lymph nodes, the PD-1/PD-L1 axis appear to take place in the local tumor microenvironment (33–35), PD-1 is expressed highly on T cells following repeated activation and chronic stimulation (36). As opposed to the draining lymph nodes, the PD-1 ligands PD-L1 and PD-L2 are more widely expressed and upregulated at effector sites of immune responses such as at inflamed tissues or the tumor microenvironment itself where they function to suppress T cell responses (36). The importance of interferon signaling in regulating the expression of PD-L1 and PD-L2, suggests that their expression patterns in advanced cancer and following CPI treatment may reflect a mechanism of primary or acquired resistance (34, 37). PD-1 and PD-L1 interaction inhibits T cell proliferation, survival, and effector function including cytokine release and tumor-targeted killing and can promote regulatory T cells differentiation (36, 38–41). Blocking this interaction via anti-PD-1 or anti-PD-L1 monoclonal antibodies restores the T cell from its “exhausted” phenotype, improves local T cells proliferation and effector function, and ultimately results in anti-tumor immunity (34).

**Figure 1:**
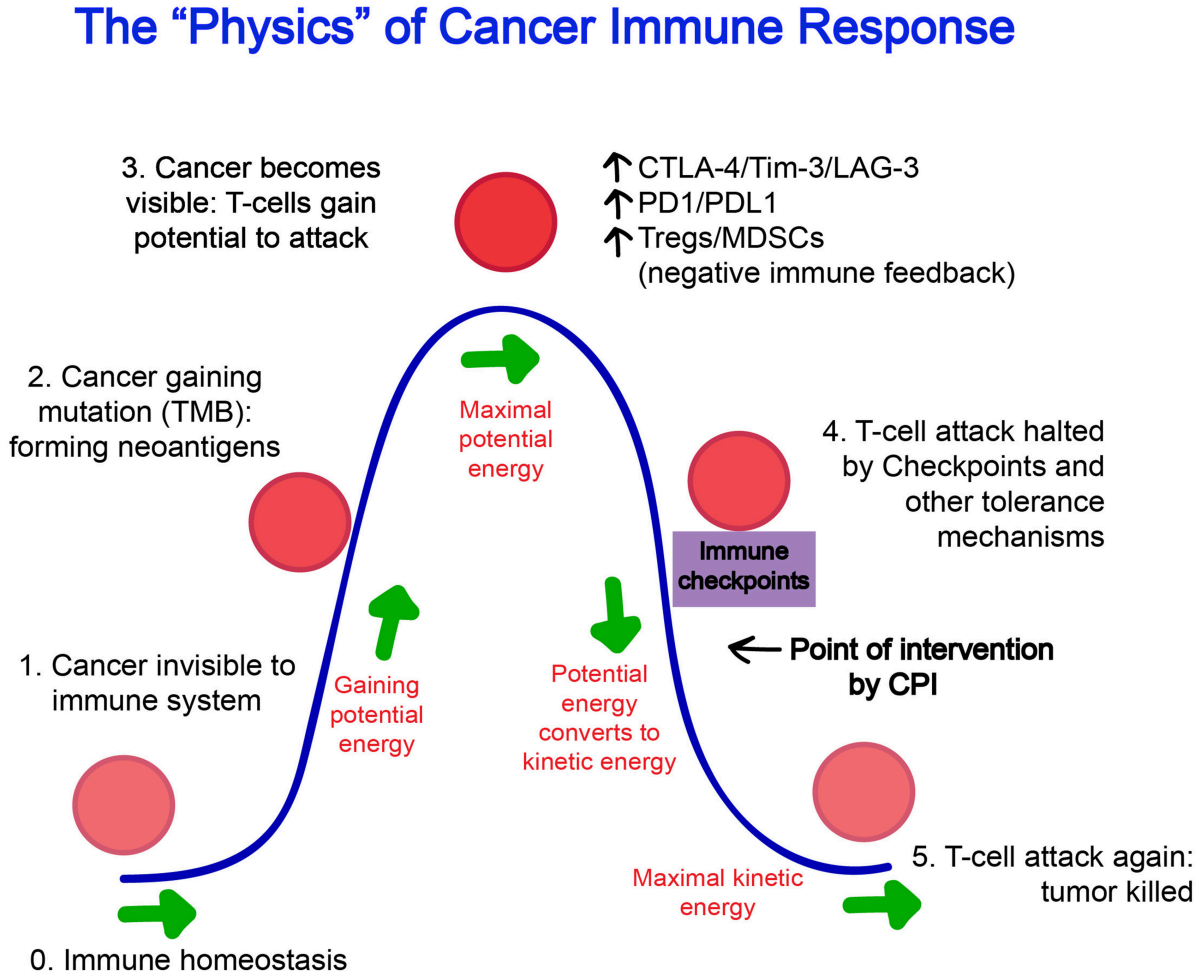
The “Physics” of Cancer Immune Response. (0) The steady state of the immune system at homeostasis is without effector cell activation and inflammation. (1) At its inception, a cancer cell may be invisible to the immune system and not trigger any response. (2) As cancer cells gain mutations over time, protein products foreign to the host are formed and these neoantigens increasingly gain recognition by the immune system. (3) “Potential energy” increases for the immune system to act, and reaches a threshold maximum where an immune attack on the cancer cells begins. (4) Naturally evolved feedback mechanisms such as T_regs_, CTLA-4, and PD-1 attempt to restore the immune system back to homeostasis and halts ongoing immune response, however, unlike infectious elements that have been cleared, the immune suppression is often premature as antigens generated from cancer cells persist with the growing tumor. This is the point of intervention where agents such as checkpoint inhibitors are thought to exert its effect. (5) After successful blockade of immune checkpoints, the “kinetic energy” of the immune system resumes and can reach its maximum and tumor can be fully eradicated.

The tumor microenvironment is a key location where tumor cells and the host immune system interact. When a cancer-associated antigen triggers an initial immune response, a series of tumor microenvironment modifications occur and impact the fate of antitumor immunity (42). The mutation-derived neoantigens are presented by the patients' major histocompatibility complex (MHC) as “foreign” antigens, recognized as such by the patients' immune system. The priming and activation of these cancer-reactive T cells is thought to occur in draining lymph nodes, generating helper, and cytotoxic T cells which traffic into the tumor, exert effector functions, and carry with them the potential to reject the tumor (43–45). At this phase, however, the pro-inflammatory environment generated by the inciting immune response favors the upregulation of reactive “shut-down” mechanisms that function to restore immune homeostasis, likely evolved to prevent aberrant immune reactions and the destruction of healthy host cells Figure 1.

These checkpoint and tolerance mechanisms often prevent successful tumor eradication, but their presence and detection in the tumor microenvironment also provide evidence that an initial immune recognition has occurred and a battle between immune cells and tumor has begun. This is the scenario in which immunotherapy can help offset the balance toward tumor killing. This principle is behind the development of PD-L1 testing as a companion predictive biomarker for PD-1/PD-L1 based CPI treatment and is currently the only commercially available FDA approved predictive biomarker for cancer immunotherapy (section Tumor mutational burden, PD-L1, and other tumor microenvironment-associated biomarkers for checkpoint inhibitor treatment; Table 1). PD-L1 expression in pre-treatment tumor or immune cells is upregulated as a consequence of proinflammatory cytokine interferon-gamma (IFN-γ) released from T cells activation, presumably by tumor-associated antigens within the microenvironment (44). The presence of this immune response is correlated with a significantly higher chance that PD-1 based treatment will work in metastatic melanoma and a variety of other tumor types (46, 47).

**Table 1:** Commercially available PD-L1 diagnostic tests.

**IHC assay**	**Assay developer**	**Companion drug**	**FDA indications**	**Expression source**	*Scoring cutoff	**Antibody clone**
Ventana PD-L1 (SP142)	Ventana	Atezolizumab (anti-PD-L1)	Bladder Cancer, NSCLC	FFPE of tumor infiltrating immune cells and tumor cells	≥10%	Rabbit SP142
Ventana PD-L1 (SP263)	Ventana	Durvalumab (anti-PD-L1)	Bladder Cancer	FFPE of tumor cells	≥25%	Rabbit SP263
PD-L1 IHC 22C3 pharmDx	Dako	Pembrolizumab (anti-PD1)	NSCLC, Gastric Adenocarcinoma, Cervical Cancer	FFPE of tumor cells and stroma	≥ 1%	Mouse 22C3
PD-L1 IHC 28-8 pharmDx	Dako	Nivolumab (anti-PD1)	NSCLC, Melanoma, SCC of Head and Neck, Bladder Cancer	FFPE of tumor cells	≥ 1%	Rabbit 28-8

* *Scoring cutoff varies by indication/study; unless otherwise indicated, reference value provided is based on sample non-small-cell lung cancer tumor cell IHC cutoffs*.

For metastatic skin malignancies, the durable response rates from checkpoint blockade are unprecedented, with PD-1 inhibitor monotherapy approaching 40% and the hallmark combination approach of dual CTLA-4 and PD-1 blockade providing objective responses approaching 60% in patients with metastatic melanoma, a cohort with a historically dismal treatment response rate and prognosis (48). Furthermore, of the patients that achieve a complete response, >90% remain disease free even with monotherapy, with patients from the largest KEYNOTE-01 (NCT01295827) study still not reaching median treatment response duration at 5 years (49). Encouragingly, the response rates in non-melanoma skin cancers follow a similar trend, with avelamub (anti-PD-L1 antibody) now approved as first-line therapy for metastatic MCC, and cemiplimab (anti-PD-1 antibody) granted FDA priority review in 2018 for SCC, with clinical trials are underway for BCC (NCT03132636, NCT02690948). Despite these advancements, the observation that durable objective response can occur in these difficult to treat metastatic solid tumors with the blockade of checkpoint pathways comes with the frustration that treatment responses and their associated irAEs are highly variable and unpredictable. Nevertheless, there are several irAES with reported correlations to clinical response, notable examples include autoimmune skin depigmentation (vitiligo) and Type 1 Diabetes (8, 50–59). The precise mechanism of irAEs, whether they are driven solely by the reduction of T cell exhaustion and aberrant activation to autoantigens via CPI treatments or by changes in the epigenetic transcriptional control in the effector T cells, is currently unknown (52). As detailed below, research efforts are rapidly underway to help uncover the determinants of anti-tumor response for immunotherapy and their associated predictive biomarkers.

## Overview of Current and Emerging Standard-of-care Treatments in Metastatic Skin Cancers

Metastatic melanoma is associated with the greatest mortality of all the skin malignancies and is often considered one of the deadliest metastatic cancers. Prior to the US FDA approval of the CPI ipilimumab in 2011, even with multi-modality systemic therapy, including surgery, radiation, and chemotherapy, the prognosis was grim, with an estimated 5-year survival of < 20% and median survival of < 1 year (60, 61). Fortunately, the standard-of-care has been shifting over the last decade, as a direct consequence of the success of CPIs. Drugs that block the PD-1 pathway (nivolumab, pembrolizumab, etc.) have been shown to provide higher response rates with a comparable durability of response and less toxicity than the cytotoxic T-lymphocyte associated protein 4 (CTLA-4) blocker ipilimumab (49, 62–64). Consequently, PD-1 blocking CPIs, with or without CTLA-4 blockade (ipilimumab) are now the preferred US FDA approved first-line therapy (Table 2). Chemotherapy, surgery, radiation, MEK inhibitors, and BRAF-mutation targeted therapy rarely produce durable responses, as mentioned above (2). They are options reserved for cases where rapid debulking and early response is necessary, often due to cancer impingement on vital structures (i.e., large brain metastasis), as they are able to achieve a faster, albeit often temporary, regression of large masses (NCCN guidelines version 3.2018). Older generation immunotherapy drugs such as systemic high-dose interleukin-2 (IL-2) or interferon alpha (IFNα) and bio-chemotherapy combinations (decarbonize, cisplatin, vinblastine, IL-2, and IFNα) are rarely recommended due to low efficacy and high toxicity, even in the adjuvant or second-line settings. While we await updated metastatic melanoma survival data in the age of modern immunotherapy, CPIs are expected to significantly improve and thus transform the standard-of-care and outlook for patients with this devastating cancer.

**Table 2:** Current status of immunotherapy drugs in metastatic skin cancer.

**Cancer type**	**Pre-immunotherapy drugs**	**Immunotherapy drugs**	**US FDA approved**
Metastatic Melanoma	Chemotherapy (i.e., Dacarbazine, Temozolomide, nab-Paclitaxel, Carboplatin); Targeted therapy (i.e., Dabrafenib, Trametinib, Combimetinib, Vemurafenib, Binimetinib)	High-dose Interleukin-2, Interferon-α (adjuvant only), Ipilimumab (anti-CTLA-4), Nivolumab (anti-PD1), Pembrolizumab (anti-PD1), Talimogene Laherparevec (oncolytic virus)	All Approved
Merkel Cell Carcinoma	Chemotherapy (i.e., Carboplatin, Etoposide, Cyclophosphamide, Doxorubicin, Vincristine); Targeted therapy (i.e., Pazopanib)	Avelumab (anti-PD-L1), Pembrolizumab, Nivolumab, Ipilimumab, Talimogene Laherparevec	Avelumab (approved), Others (in clinical trial)
Squamous Cell Carcinoma	Chemotherapy (i.e., Cisplatin, Doxorubicin, Bleomycin, Fluorouracil); Targeted therapy (i.e., Cetuximab, Panitumumab)	Cemiplimab (anti-PD1), Nivolumab, Pembrolizumab, Talimogene Laherparevec	Cemiplimab (under FDA priority review), Others (in clinical trial)
Basal Cell Carcinoma	Chemotherapy (i.e., Cisplatin, Doxorubicin, Paclitaxel); Targeted therapy (i.e., Sonidegib, Vismodegib)	Ipilimumab, Nivolumab, Pembrolizumab, Talimogene Laherparevec	All in clinical trial

BCC, SCC, and mMCC comprise the vast majority of non-melanoma skin cancers. Like melanoma, at the early stage, local and surgical therapies are often curative and systemic therapies are only considered in settings where the tumor is unresectable or has metastasized. When disseminated, these cancers are comparable in mortality to metastatic melanoma and are notoriously difficult to treat. Similarly, the paradigm for their systemic treatment has shifted away from conventional cytotoxic modalities, which are often ineffective, to CPIs. In 2017, the FDA accelerated approval avelumab, a PD-L1 blocker, as the first-ever and only FDA approved drug for the aggressive metastatic MCC highlighting this trend (Table 2). For metastatic BCC and SCC, currently approved targeted therapies such as hedgehog pathway inhibitors vismodegib and sonidegib for BCC and epidermal growth factor receptor pathway inhibitors (cetuxmimab, pantimumumab, gefitinib, erlotinib) for SCC, although with proven response rates, face the challenge primary or acquired resistance in their respective pathways and have response durations that are often short-lived (65–69). Due to their shared feature of high tumor mutational burden (70–72), PD-1 blockade has demonstrated significant numbers of durable responses for SCC and BCC (73–76) and may likely be considered as a new standard-of-care for these indications.

As the standard-of-care for these different types of metastatic skin cancers converge on CPIs, the treatment decision process of BCC, SCC, and MCCs will likely share the clinical experience gained from MM. Some major challenges remain in the clinical deployment of CPIs for metastatic skin cancers: (1) The response rate for PD-1 inhibitor monotherapy tops out at around 40%, and while in a recent phase 3 trial combination therapy with PD-1 and CTLA-4 blockade found the response rate closer to 60%, it carries a significantly more serious toxicity profile leading to the frequent need for early treatment discontinuation (6, 48, 63, 77). (2) The general unpredictability of responses/toxicities of CPIs makes monitoring challenging and the determinants on how to shift the balance toward more response and less toxicity are largely unknown. (3) What happens if there is a relapse? Are you able to re-start CPI treatment again and get a response? Does resistance to one CPI treatment pathway infer resistance to all? (4) When to stop treatment? The high cost associated with CPIs makes it challenging to continue treatment indefinitely (78), and despite some reports of prolonged efficacy after discontinuation, the optimal time to stop treatment to ensure sustained complete response is currently unknown (49). In practice, in responding patients, many clinicians continue treatment until complete response plus at minimum 1 year after, but there have been no randomized clinical trials designed to address this important question. These and other considerations necessitate the interest and resources dedicated to better the mechanistic understanding of how CPIs work as well as develop biomarkers for treatment so that we can more rationally and efficiently deploy them. Ideally, we can improve upon current overall response to CPIs while lessening toxicity, but until then, predictive biomarkers could help target PD-1 monotherapy to those who have a higher probability of response and help identify those for whom a more aggressive combination of immunotherapy is needed.

## Tumor Mutational Burden, PD-L1, and Other Tumor Microenvironment-associated Biomarkers for Checkpoint Inhibitor Treatment

TMB is a mechanistically rational biomarker for CPI treatment efficacy. As the skin is the first line of host defense against environmental assaults, providing the physical barrier against the mutagenic effects of UV radiation, invasion of viral pathogens, and colonization of commensal bacteria, it stands to reason that this organ system and cancers that arise from it should accumulate higher antigenic mutational burden for T cell detection (79, 80). Indeed, all metastatic skin cancers have demonstrated significant responses to CPI treatment (9), and TMB analysis has shown that melanoma, SCC, BCC, and MCC, typically harbor mutational and antigenic loads at the higher end of the mutational spectrum (8, 9, 70–72). Over time, the environmental assaults on the skin accumulate mutations and result in a “tug-of-war” between oncogenic driver mutations that create cancerous lesions and immunogenic passenger mutations that may then cause its regression from immune recognition (81–83). In theory, high tumor mutational burden provides a source of tumor-associated peptides that become neoantigens which trigger the initial differential T cell recognition of cancer cells from normal cells and provide the requisite anti-tumor immune response that allows patients to respond to immune- based treatments (79, 84). Clinical evidence is supportive for TMB as a biomarker of checkpoint response, as there is a correlation with the number of mutations and the chance such a mutation will provide a productive immunogenic antigen. In addition, clinical observations correlating cancers with high tumor mutational load with a better checkpoint blockade response raised the initial possibility of an association (10, 11). This hypothesis was further validated after reports that DNA mismatch-repair deficiency associated cancers (microsatellite instability-high) harbor some of the highest known tumor mutational burdens. Patients with this class of tumors have one of the highest response rates to PD-1 inhibitors (85, 86). These findings eventually led to accelerated FDA approval in 2017 for all cancers that are microsatellite instability-high.

PD-L1 is currently a U.S. FDA approved biomarker for PD-1 based CPI therapies. In general, patients with higher PD-L1 levels by immunohistochemistry have higher chances of responding to PD-1 based CPI therapy (87, 88). However, the predictive value for response is more accurate for patients with lung cancer than for skin cancers such as melanoma (89, 90), as durable responses to PD-1 CPI treatment has been observed in some melanoma patients with low expression of PD-L1. In terms of correlation to overall survival, interestingly, the majority of prior reports (before the wide usage of immunotherapy) suggest that high expression of PD-L1 correlates with shorter survival, even in settings of a high tumor mutational burden (16, 91–94). The paradoxical relationship between lower historical overall survival and higher PD-1 therapy response rate can be reconciled with the explanation that tumors with high tumor mutational burden generally leads to higher local expression of PD-L1, because more T cells sensitive to the tumor are likely to be present to be activated. On the other hand, highly mutated tumors would also have a higher potential to evolve driver mutations or pathways for adaptive resistance (81, 95–97) (Figure 1), therefore bypassing mechanisms of tumor rejection. Thus, while the value of PD-L1 as a biomarker for predicting better PD-1 therapy response is established, its use as a predictor for overall survival remains controversial and is to be determined in the era of immunotherapy. Neither the use of TMB nor PD-L1 is perfect, and the use of the combination of TMB and PD-L1 to identify likely responders to CPIs may be costly. Until more effective biomarkers are available, one clinical strategy for their use in skin cancers that has demonstrated some success is to utilize TMB and PD-L1 together as negative instead of positive predictors of response- when a low TMB and PD-L1 immune signature is present, this indicates that stronger immunotherapy such as PD-1 and CTLA-4 combination therapy should be utilized (98).

As more sophisticated means of patient immune monitoring both on and off therapy are being developed, other potential biomarkers are being investigated. For example, various other T cell activation and exhaustion markers including inducible T cell costimulatory (ICOS), 4-1BB (CD137), PD-1, CTLA-4, lymphocyte activation gene 3 (LAG-3), T cell immunoglobulin mucin-3 (Tim-3) and CD39, whether in circulation or within the tumor microenvironment, are now being correlated with response rates (46, 99–104) These, and other immune activation related biomarkers are biological surrogate markers that collectively signify an active immune response within the tumor microenvironment for which drugs such as CPIs can potentially take advantage, and may add to the predictive value and reliability of tumor mutational burden and PD-L1 expression (Figure 1). For biomarkers that are not directly associated with immune activation, serum lactate dehydrogenase level assessments have traditionally been made in the clinic as a surrogate indicator of disease progression and poor prognosis in melanoma, based on the rationale that lactate dehydrogenase is released upon cell damage and death associated with elevated tumor burden. Low baseline levels further demonstrate a favorable response correlation with CPI usage (105). Additionally, several novel non-immune activation related biomarkers of CPI response have been proposed, and include total tumor volume (101, 106), the presence of liver metastasis (98, 107), MHC protein expression (108), and the composition of the gut microbiome (109–112). Total tumor volume and liver metastasis assessment offer the benefit of cost-effectiveness and easier accessibility in clinical use, as they can be readily identified with standard-of-care imaging. Both high tumor volume and liver metastasis have independently been reported to result in unfavorable alterations in systemic T cell profiles (98, 101), suggesting yet-to-be discovered mechanisms of tumor immune regulation. Gut microbiota composition of certain bacterial strains and overall microbiome diversity appear to correlate with response rates (110–112), but larger and more geographical and demographically diverse studies are likely needed before conclusions can be finalized to influence clinical practice. Finally, the accumulation of the variety of rationally developed biomarkers may eventually contribute to patient-individualized “immunoscoringm,” a promising precision-medicine strategy that may be immunotherapy-focused and disease-agnostic, and effectively allow for constant updates and dynamic improvements in the fast-paced predictive biomarker field (113).

## Cytokines and Chemokines as Prognostic Indicators for Skin Cancer

Cytokines and chemokines influence a complex network of regulation regarding immune cell function and trafficking. They can be divided readily into pro-inflammatory, anti-inflammatory, mitogenic, and chemotactic subgroups and are capable of contributing to particular functions dependent on their microenvironment. In addition to these properties, cytokines and chemokines and their receptors play a part in biological functions relevant to oncogenesis, including tumor cell proliferation, protease induction, and angiogenesis. Thus, cytokines and chemokines can facilitate immune-mediated tumor rejection or promote tumor progression as well as metastasis. These dichotomous associations and their underlying mechanism have become an area of active research not only for biomarker development but also for novel therapeutic targeting. Assessment of cytokine and chemokine profiles within the tumor microenvironment or in circulation, may greatly increase the resolution of our understanding of the status of the immune response to the tumor. The ability to tease apart the delicate balance of effector vs. regulatory elements in the tumor microenvironment will be essential to patient therapeutic design targeting a variety of critical aspects of the immune axis that could be dysregulated in cancer disease progression.

A number of chemokines and cytokines have been investigated for their roles in skin malignancies. Pro-inflammatory cytokines including TNF-α, Interleukin-16 (IL-16), IL-17, IL-21, IL-22, and IL-23 have all been associated with tumorigenesis and an inflammatory environment. Cytokines like IFN-γ and IL-12 are cytotoxic T cell related cytokines associated with mounting a cytolytic immune response. Their anti-tumor response is well-characterized in limiting skin cancer development (114–117). Immune-modulating cytokines including IL-10 and TGF-β are reported to oppose anti-tumor response since they are made predominantly by T regulatory cells to suppress cytotoxic responses against the skin malignancies (114, 118–120). However, like all cytokines, their roles are context-specific. For example despite circulating IL-10 levels have been associated with poor patient prognosis in a number of cancer settings, yet IL-10 expressing CD8 T cells confer a positive prognosis in patients with lung cancer (121–123). Chemokines, similarly, have long been associated with the recruitment of cells into the skin and can be sub-grouped as homeostatic or pro-inflammatory chemokines. Homeostatic chemokines are expressed constitutively and play key roles in normal leukocyte development and trafficking, pro-inflammatory chemokines are inducible and are responsible for trafficking to inflammation associated events (124). The chemokine receptor CXCR3 is expressed on inflammatory infiltrates and mediates attraction to the IFN-γ-inducible chemokines CXCL9 (or MIG), CXCL10 (or IP-10), and CXCL11(I-TAC) into the skin (125–129). These pathways of recruitment are also capable of regulating immune cell differentiation and activation, favoring Th1 and IFN-γ-production, respectively. The IFN-γ/CXCL9, CXCL-10, CXCL-11/CXCR3 axis can be a double-edged sword in cancer, promoting an anti-tumor effect but also increasing the capability of cancer cell proliferation, angiogenesis and metastasis (125). CXCL1 (melanoma growth-stimulatory activity/growth-regulatory protein α) and CXCL8 (IL-8) have important roles in inflammation, angiogenesis, tumorigenesis, and wound healing (130). However, these pathways can also be hijacked in cancer, promoting inflammation, inducing angiogenesis, tumor growth, and metastasis (131). For melanoma the expression of chemokine receptors CCR10, CXCR4, and CCR7 have been attributed to tumor escape and preference for metastasis (132). A number of reviews have addressed the importance of chemokines in skin cancer growth and metastasis (17–22, 133). Fewer, if any, have addressed their potential as biomarkers for prognosis or treatment outcomes.

## Cytokines and Chemokines as Biomarkers for Checkpoint Inhibitor Treatment in Malignant Skin Cancers

Although limited, a number of groups that have studied cytokines and chemokines as biomarkers for predicting clinical outcomes to CPI therapies for skin malignancies. The prototypical T cell growth factor, IL-2, and the major anti-inflammatory cytokine, IL-10, have yielded surprisingly little correlation with actual clinical response rates, possibly due to the transient and locally expressed nature of these cytokines (88, 134). Available research, with many focused on patients treated with CTLA-4 inhibitors that were approved much earlier, with their main findings and predicted clinical outcomes are summarized in Table 3 and discussed below.

**Table 3:** Chemokine & Cytokine biomarkers investigated for CPI treatment outcomes.

**Treatment**	**Biomarker category**	**Biomarker**	**Associated outcome**	**Study summary**	**References**
**MELANOMA**					
CTLA-4	Blood soluble immune factor	IL-17, TGF-β & IL-10	Baseline levels predict toxicity and relapse	33 patients; blood and serum; baseline and 6 weeks following treatment	(135)
CTLA-4	Blood soluble immune factor	IL-6	High levels above media associated with treatment failure	40 patients; blood and serum taken at baseline and following up to 4 treatments	(136)
CTLA-4	Blood soluble immune factor	CXCL11 & sMICA	High baseline levels associated with poor overall survival to treatment	137 patients; blood and serum; independently validated in different cohort; baseline levels	(137)
CTLA-4	Blood soluble immune factor	IL-8	Decreases in serum levels in responders vs. increased levels in non-responders	8 patients; blood and serum; same response correlated with iBRAF treatment responses	(138)
PD-1	Blood soluble immune factor	IL-8	Early changes (decrease) were strongly associated with response	29 patients; blood and serum; independently validated in cohort of 12 melanoma and 19 NSLCL patients	(139)
PD-1	Blood soluble immune factor	IFN- γ, IL-6 & IL-10	Higher baseline levels were found in patients with objective tumor response compared to those with progression	37 patient; blood and serum; baseline and day 43	(134)
PD-1	Blood soluble immune factor	IL-9 & TGF-β	Increase frequency of IL-9 producing CD4 T cells and increased pre-treatment TGF-β serum levels in responders	46 patients; 18 responders and 28 non-responders; pre and post treatment (3 infusions)	(120)
PD-1	Blood soluble immune factor and Tumor tissue gene expression	IFN- γ, IL-18, CXCL11 & IL-6	Serum changes were observed. Increased IFN- γ genes in pretreatment tumor biopsies associated with response	Blood and serum samples taken before and after treatments	(140)
CTLA-4	Tumor tissue gene expression	IFN-γ, CCL4, CCL5, CXCL9, CXCL10, CXCL11, IDO1, GBP1 and class II MHC molecules	Higher baseline levels of immune-related genes predicted clinical response	45 patients; tumor biopsy; pre and post treatment	(141)
CTLA-4	Tumor Whole-exome sequencing	IFNGR1, IFNGR2, JAK2, IRF1, IFIT1, IFIT2, MTAP, miR3, SOCA1 & PIAS4	Tumors that are resistant to treatment contain genomic defects in the IFN- γ pathway genes	12 non-responders and 4 responders; Tumor samples	(142)
CTLA-4 followed by PD-1	Tumor tissue gene expression	GZMA, GZMB, PRF1, HLA-DQA1, HLA.DRB1, IFNG, STAT1, CCL5, CXCL9,−10, - 11, ICAM1-5 & VCAM-1	Active immune signature in early tumor samples were highly predictive of response	5 responding patients and 6 non-responders following PD-1 treatment; tumor samples	(143)
PD-1	Tumor Whole exome sequencing	JAK1 & JAK2	Tumors that are resistant to treatment contain genomic defects in the IFN- γ pathway genes	4 patients; initially had an objective response to treatments but went on to have disease progression	(144)
PD-1	Tumor tissue gene expression	IFNG, STAT1, CCR5, CXCL9, CXCL10, CXCL11, IDO1, PRF1, GZMA, MHCII HLA-DRA, CXCR6,	Immune-related signature using RNA from baseline tumor samples which correlated with clinical benefit	19 patients; tumor biopsies prior to treatment; validated in 62 melanoma patients	(145–147)
		TIGIT, CD27, CD274 (PD-L1), PDCD1LG2 (PD-L2), LAG3, NKG 7, PSMB10, CMKLR1, CD8A, IDO1, CCL5, CXCL9, HLA.DQA1, CD276, HLA.DRB1, STAT1 & HLA.E			
PD-1	Tumor tissue gene expression	CD3D, CD3E, CD3G, CD247, ZAP70, CD2, CD28, ICOS, IL12Rb1, CXCR3, STAT4, PRF1, IFNG, CD8A, CD8B, GZMM & FLTSLG	Immune-related signature from baseline tumor samples where associated with non-progressive disease and progression free survival	25 patients; tumor biopsies prior to treatment	(148)
PD-1	Tumor tissue gene expression	IFNG, IDO1, CXCL9, CXCL10 & CXCL11	Strong positive correlation between IFN-γ and IFN-inducible genes is associated with response and prolonged overall survival	21 melanoma patients; tumor biopsies prior to treatment; 17 NSCLC patients	(149)
**SCC**					
PD-1	Tumor tissue gene expression	CXCL9, CXCL10, IDO1, IFNG, HLA-DRA & STAT1	IFN- γ signature may be associated with clinical response	56 patients; tumor RNA extracted from FFPE slides	(150, 151)
PD-1	Tumor tissue gene expression	CXCR6, TIGIT, CD27, CD274 (PD-L1), PDCD1LG2 (PD-L2), LAG3, NKG 7, PSMB10, CMKLR1, CD8A, IDO1, CCL5, CXCL9, HLA.DQA1, CD276, HLA.DRB1, STAT1 & HLA.E	Immune-related signature using RNA from baseline tumor samples which correlated with clinical benefit	Validated in 43 HNSCC patients.	(145)
PD-1	Tumor tissue gene expression	CD3D, CD3E, CD3G, CD247, ZAP70, CD2, CD28, ICOS, IL12Rb1, CXCR3, STAT4, PRF1, IFNG, CD8A, CD8B, GZMM & FLTSLG	Immune-related signature using RNA from baseline tumor samples where associated with non-progressive disease and progression free survival	5 patients; tumor biopsies prior to treatment	(148)

HNSCC, Head and Neck SCC; NSCLC, Non-small cell lung cancer; FFPE, Formalin-Fixed Paraffin-Embedded

### TGF-β and IFN-γ

Tarhini et al. (135) evaluated circulating serum levels of cytokines, chemokines and a number of growth factors in response to CTLA-4 therapy (ipilimumab) finding a strong correlation between baseline levels of IL-17 and irAEs, and the combination of TGF-β and IL-10 baseline levels were predictive of relapse against therapy (135). Interesting, TGF-β has also been proposed as a serum marker of response to PD-1 (nivolumab) therapy in a cohort of MM patients by Nonomura et al. (120), with significantly increased pre-treatment levels, but not post-treatment levels, reported between responders and non-responders (120). The group found increased levels of newly characterized CD4 population, Th9 cells, that are generated in the presence of TGF-β and IL-4, suggesting that the blockade of PD-1/PD-L1 axis promoted Th9 cell differentiation, which in turn suppressed melanoma progression and increases cytotoxic activities of CD8 T cells (120). Although conflicting, the presence of TGF-β is reflective of a reduced immune response and therefore more likely to represent resistance to CPI treatments (152–154). In melanoma TGF-β production is positively correlated relative to disease progression, acting as a tumor promoter rather than a suppressor, and negatively regulating the activity of T cells by blocking IL-2 production (119).

The importance of an immune-active tumor microenvironment in clinical responses for CPI treatment has been shown in a number of melanoma patient cohorts. Early work performed Herbst et al. (140) to understanding predictive correlates of response to PD-1 therapy (MPD3280A) examined blood-based biomarkers. However, despite increases in IL-18, CXCL11 (ITAC), and IFN-γ found during the initial stages of treatment, there was no correlation to patients' long-term response and outcomes (140). Examining tumor tissue gene expression, however, they did find a number of genes associated with enhanced T-effector cell activity in pre-treatment responsive tumors compared to non-responsive tumors, including *IFN-*γ*, IDO-1*, and *CXCL9* (140). In 2012, Ji et al. reported a phase II clinical trial in which 45 melanoma patients were treated with CTLA-4 (ipilimumab), and treatment was found to induce IFN-γ-inducible genes *IDO1, GBP1*, and class II MHC molecules and a number of Th1-associated marker genes *IFN-*γ*, CCL4, CCL5, CXCL9, CXCL10*, and *CXCL11* in patients with a clinical response (141). The importance of IFN-γ gene signatures for response has been published similarly by Ribas et al. (146) in a test cohort of 19 melanoma patients treated with PD-1. Comparing the gene signature of responders vs. non-responders revealed a number of top-ranking genes that were strongly associated with IFN-γ signaling and correlation with IFN-γ expression. Their preliminary IFN-γ gene signature included *IFNG, STAT1, CCR5, CXCL9, CXCL10, CXCL11, IDO1, PRF1, GZMA*, and MHCII *HLA-DRA* and an expanded immune signature of genes related to cytolytic activity (e.g., granzyme A/B/K and PRF1), cytokines and chemokines (e.g., CXCR6, CXCL9, CCL5, and CCR5), T cell markers (e.g., CD3D, CD3E, CD2, IL2RG), NK cell activity (e.g., NKG &, HLA-E), antigen presentation (e.g., CIITA, HLA-DRA) and other immunomodulatory factors (e.g., LAG3, IDO1, and SLAMF6) were able to differentiate between objective responses and non-responders. Additionally, the group was able to further refine their IFN-γ and expanded immune gene signatures within another cohort of 62 melanoma patients (145–147), in which the baseline immune-related tumor samples correlated with clinical benefit from treatment. The importance of activated Th1 and cytotoxic T cells in mediating CPI mediated tumor responses irrespective of the tissue environment was also used to test and further refine the gene signature, with the group finding these signatures capable of associating with treatment response to pembrolizumab in HNSCC and gastric cancer. Furthermore, the final T cell-inflamed gene expression profile consisting of 18 genes, (*CXCR6, TIGIT, CD27, CD274* (PD-L1), *PDCD1LG2* (PD-L2), *LAG3, NKG* 7, *PSMB10, CMKLR1, CD8A, IDO1, CCL5, CXCL9, HLA.DQA1, CD276, HLA.DRB1, STAT1*, and *HLA.E*), was tested against 9 tumor types (KEYNOTE-012; bladder, gastric, HNSCC, and triple-negative breast cancer, KEYNOTE-028; anal canal, biliary, colorectal, esophageal, and ovarian cancer) with most of the gene signature positive association with response. The data present by Ayers et al. (145) highlights the importance of the T cell-inflamed microenvironment common to patients who respond to anti-PD-1 CPI treatment regardless of the tumor type or tissue type and also proposes a difference between resistance mechanisms between non-responding patients that lack T cell inflammation and those who have infiltration but lack clinical response (145).

Chen et al. (143) followed a unique set of melanoma patients that were initially treated with CTLA-4 followed by PD-1 at progression, identifying a unique immune signature in responders not evident in non-responding patients significant after 2 to 3 cycles of treatment (143). Within the 411 significantly differentially expressed genes of responders where a number of cytolytic makers (i.e., GZMA, GZMB, and PRF1), HLA molecules (e.g., HLA-DQA1 HLA.DRB1) IFN-γ pathway genes (e.g., IFNG, STAT1), chemokines (e.g., CCL5, CXCL9,−10, and 11) and select adhesion molecules (i.e., ICAM1-5 and VCAM-1). The dataset also allow for the comparison of pretreatment samples and after treatment fold changes clearly differentiating responders from non-responders, regardless of the pretreatment with CTLA-4. These finding clearly support the assessment of early immune responses can be highly predictive of overall response to therapy. However, it should also be noted that these on treatment changes maybe associated solely with treatment and not associated with a mechanism of response. In additional Chen et al. also highlight an angiogenic phenotype in non-responding patients (decreased *VEGFA*), downregulation of antigen processing and presentation (*HLA* genes) and defects in the interferon signaling pathways, all of which are consistent with a number of previously published works (142, 143, 155–159).

Prat et al. (148) evaluated pre- and post-PD-1 (pembrolizumab or nivolumab) treated tumor samples from a range of cancers, including melanoma patients. Twenty three immune related genes or signatures where identified to be associated with non-progressive disease and progression free survival, these included genes associated with the formation of the TCR complex (e.g., *CD3D, CD3E, CD3G, CD247*, and *ZAP70*), co-stimulatory factors (e.g., CD2, CD28, and ICOS) and genes involved in differentiation (e.g., *IL12Rb1, CXCR3*, and *STAT4)*. In addition to apoptosis pathway genes involving granzyme A, B and perforin 1, T cell receptor signaling (e.g., CD8A, CD8B, and IFN-γ), cell adhesion molecules (e.g., CD4, CD86, and integrinβ2), a number of toll-like receptors (TLR1,4, 7, and 8) and checkpoint molecules PD-1, PDL-2, and LAG3. These signatures were found across all the different cancer types assessed and 12 signatures tracking immune cells including CD8 T cell associated genes PRF1, CD8A, CD8B, GZMM, FLTSLG, CD4 T cell activated genes IL26 and IL17A as well as NK cell and B cell genes and PDL-1, PD1, and CTLA4 and IFN-signaling pathway activation (148). These findings are in support of the Ribbas et al. (146), Ayer et al. (147), and Ayer et al. (145) T cell and IFN activation predicting response to the PD-1/PD-L1 treatment across multiple tumor types and tumors with strong pre-treatment immunity.

Gao et al. (142) used whole-exome sequencing of tumor tissues to show that tumors that are non-responsive to CTLA-4 (ipilimumab) therapy have defects in IFN-γ pathway via significantly higher somatic mutations including copy-number variations and single-nucleotide variants in IFN-γ signaling genes including *IFNGR1, IFNGR2, JAK2, IRF1, IFIT1, IFIT2, MTAP*, and *miR31* and amplification of IFN-γ suppressor genes (*SOCA1* and *PIAS4*) compared to responders (142). Similarly, Zaretsky et al. (144) published a similar finding in melanoma patients treated with anti-PD-1 antibody (Pembrolizumab) finding that loss of function mutations within the IFN receptor associated Janus kinase 1 (JAK1) or Janus kinase 2 (JAK2) lead to lack of response to IFN-γ leading to acquired resistance to PD-1 blockade (144). However, both cohorts of patients were small, 16 and 4, respectively, and require validation in larger cohorts of patients and treatments. Similar observations have been previously published by Tumeh et al. (45) in a MM patient cohort treated with anti-PD-1 (pembrolizumab), with baseline and post-dosing biopsies for phospho-STAT1 (pSTAT1), an immediate downstream effector upon IFN-γ binding (45). Proposing that the PD-L1 tumor expression can be linked to interferon production within the tumor microenvironment via T-cell recognition, patients that were found to have a response to therapy had significantly higher expression of pSTAT1 at baseline and during treatment compared to progression group (45). Highlighting again the importance of a pre-existing tumor immune response in patient outcomes.

Recently, a retrospective study conducted in melanoma samples found that significantly longer progression-free survival and overall survival were observed in patients with pre-treatment high IFN-γ expression (149). High pre-treatment expression of IFN-γ-inducible genes (e.g., IDO1, CXCL9, CXCL10, and CXCL11 among others) was associated with response and prolonged overall survival for anti-PD-1 (pembrolizumab) treatment of melanoma (149). In a cohort melanoma patients treated with anti-CTLA-4 (ipilimumab; NCT00094653) Koguchi et al. (137) found elevated pre-treatment serum levels of CXCL11 and a soluble MHC class I polypeptide-related chain (sMICA) to be strong predictors of poor survival benefit, this finding was confirmed in an independent cohort of melanoma patients treated with ipilimumab (137). This finding is in contrast with tumor tissue samples that favor CXCL11 as a biomarker of T cell infiltration and therefore a favorable prognostic marker, as mentioned above. CXCL11 also has distinct immunoregulatory functions through the higher affinity to its receptor CXCR3 (125). CXCL11 is also capable of binding to CXCR7, more commonly associated with tumor growth, making its functional role controversial (125, 137, 149). In addition, the contrast in findings between protein in the serum to mRNA levels in the tissue microenvironment suggests that parallel analysis of both serum and tissue, and protein and mRNA, maybe required further clarify these differences (137).

Despite strong preclinical support of checkpoint inhibitor for the treatment of non-melanoma skin cancers, little is still known about blocking the PD-1/PD-L1 and CTLA-4 pathways in patients. A number of single case studies have been published on the use CPI in the treatment of these cancers, however due to patient numbers investigation into biomarkers for predicting treatment response has been limited. Chow et al. (150) were the first to clinically assess the use of PD-1 and PD-L1 CPIs in head and neck SCC. Keynote-012 stratified patients to assess the response of PD-1 (pembrolizumab) on PD-L1-positive head and neck SCC patients (150, 151). Using the identified gene signature (*CXCL9, CXCL10, IDO1, IFNG, HLA-DRA*, and *STAT1*) that was identified in the melanoma cohort and validated in a head and neck SCC cohort (146, 147), all six of the IFN-γ-related genes were statistically higher in responding patients compared to non-responders. However, it should be noted that the study only included PD-L1 positive patients. Further work is needed to elucidate the interaction with PD-L1 and an IFN-γ gene signature (151). Despite these initial findings, little has been published regarding biomarker discovery, outside of HPV status and PD-L1 expression (150, 160–163). Tumor tissue gene expression of similar IFN-γ gene signatures identified in independent melanoma cohorts has also been correlated with clinical benefit from anti-PD-1 treatment in a small number of head and neck SCC patient cohorts (145, 148).

The use of CPI therapies for the treatment of BCC are currently under clinical trial. A few promising case studies have been published recently (73, 76, 164) with treated efforts based predominately on the success of CPIs in similar tumors with high mutational burdens, and from the finding that ~90% of BCCs stain positively for PD-L1 expression (165). Recently, Lipson et al. (75) correlated the expression of PD-L1 in the tumor microenvironment with predictability for objective responses in PD-1-pathway-directed therapies (75). It has also been established that BCCs undergoing spontaneous regression (presumably immune-mediated) contain elevated levels of Th1 cytokines (e.g., IFN-γ) and infiltrating activated T cells (166, 167). Additionally, treatments that have focused on interferon therapy have shown efficacy in BCC (168). MCC is a rare but aggressive skin cancer, with several published studies finding expressed PD-L1 in the tumor and also PD-1 expression on tumor-infiltrating lymphocytes (159, 169–172). These observations provide a strong rationale for assessing CPIs on patients with advanced disease that has few therapies to extend survival. Current clinical trial by Nghiem and colleagues treated patients with PD-1 (pembrolizumab), response to treatment did not correlate with PD-L1 expression, so too in both virus negative and positive cancers (172).

Although strongly biased by melanoma patient data, there is strong evidence supporting the importance of an active immune microenvironment for successful CPI therapy outcomes in metastatic skin cancer. IFN-γ production by activated T cells is capable of multitude of downstream affects, including activation of dendritic cells and macrophages (like, STAT1 and CMKLR1), which in turn produce their own chemokines and cytokines (like, CCL5, CXCL9,-10,-11) to further recruit CD8 T cells. Upregulation of co-stimulatory molecules (CD27) and effector molecules (IFN-γ, perforin, and granzymes) further contribute to the immune response. IFN-γ induces upregulation of HLA molecules and other pathway associated with antigen processing and presenting. IFN-γ also drives upregulation of PD-L1 and PD-L2 on the surface of macrophages, DCs and tumor cells. Other checkpoint molecules like IDO1, TIGIT and LAG3 are similarly associated with T cell activation and IFN-γ signaling to help restrain antitumor responses (145). The use of gene expression signature highlights the complexity within the immune response associated with CPI treatment and response and suggests the importance of multiple cell types within the tumor microenvironment. These gene expression profiles provide an interesting insight into the immune responses to CPI therapy and highlight a number of possible targets that can be manipulated for better therapeutic outcomes.

### IL-6 and IL-8

IL-6 is a pleiotropic cytokine that is associated similarly with disease pathogenesis and metastasis and has already been published as a serum marker for melanoma patients. IL-6 has also been linked to IL-10 production within the melanoma tumor microenvironment. For head and neck SCC, BCC, and mMCC high IL-6 levels are also associated with a poor prognosis and treatment resistance (173). The expression of PD-L1 has also been linked to IL-6 and IL-10 cytokines, which may explain their role in tumor progression and as a marker of non-response. Bjoern et al. (136) found MM patient cohort following CTLA-4 treatment (ipilimumab) who had lower serum baseline levels and lower levels at the 4th dose of pro-inflammatory cytokine IL-6 responded better to therapy (136). This observation of serum IL-6 as a poor prognostic biomarker for MM immunotherapy responses has been previously suggested in IL-2-based and bio-chemotherapy therapies (174, 175). Pre-treatment serum levels of IL-6, IFN-γ, and IL-10 were found to be significantly higher in patients with objective tumor responses in a cohort of phase 2 advanced melanoma patients treated with PD-1 (nivolumab) in comparison to non-responders with progressive disease (134). The increased cytokine levels were also positively correlated with each other, suggesting both a spontaneous activation and suppressive response at the same time (134). In addition, the group found increases in CXCL9 and CXCL10 between pre-treatment and post-treatment (Day 43) serum samples in response to treatment, postulated to be associated with IFN-γ production by activated T cells in the blood (134).

IL-8 serum levels have been proposed to reflect tumor burden and decreased levels in a small cohort of MM patients during treatment with CTLA-4 (ipilimumab) correlated with patient benefit from treatment (138). Sanmamed et al. found that serum IL-8 levels are a consequence of tumor burden in a number of cancer types including melanoma and could be monitored to predict response to BRAF inhibitors and correlated to overall survival (138). In addition, the authors recently published that changes in serum IL-8 levels reflect tumor response to PD-1 treatment (nivolumab or pembrolizumab) in MM patients in both identification cohort and independent validation cohort (139). Decreases in IL-8 serum levels were found at patient's best response, additionally, they were able to distinguish pseudo-progression (decreased) and non-responders (increased) and also monitor responses via IL-8 serum fluxes (139). IL-8 has been similarly implicated as a biomarker for head and neck SCC patients receiving chemoradiation with or without novel hypoxic cytotoxins (176). A recently published case report describing a recurrent head and neck SCC patient on PD-1 (nivolumab) treatment found increased levels of IL-8 and IL-6 following treatment (from pre-treatment levels) and decreased levels of IL-10 and CXC3C1, the patient was found to have progressive disease (177), this cytokine and chemokine profile is constant previously published work from a cohort of melanoma and non-small-cell lung cancer patients (139). The role of IL-8 and its receptor CXCR2 in tumor development and progression has been well-documented in a wide range cancer cells including melanoma, SCC, BCC, and MCC (18, 146, 178–182). IL-8, and its receptor CXCR2, are poised to be examined as potential biomarkers in both BCC and mMCC treated with CPIs. The induction of pro-inflammatory cytokines like IL-6 and IL-8, appear strong indicators of unsuccessful CPI therapy and highlight the need for biomarkers for patients who will not respond to treatment as well as for those who will.

## Predictive Biomarkers for Immune-Related Adverse Events

As stated above, checkpoint inhibitor therapy can be associated with severe or even life-threatening irAEs. Generally, irAEs occur within weeks to 3 months after initiation of treatment but have been documented to occur months after discontinuation of treatment. Fatigue is the most common irAE reported following treatment with either anti-CTLA-4 and anti-PD1/anti-PDL-1 antibodies and can range up to 50% (183). For PD-1 based therapy, the next most common (10–20%) are grade 1-2 skin rash, transaminitis, arthralgia, colitis, low-grade fevers, thyroiditis, and other endocrinopathies. For CTLA-4 based therapies, the next most common (20–30%) are grade 1–2 colitis, anemia, transaminitis, skin rash, arthralgia, and low-grade fevers(184). Grade 3–4 irAEs are rare for PD-1 based therapies but are more common in CTLA-4 based or combination CTLA-4 plus PD-1 therapies, and are mostly commonly colitis, transaminitis, and endocrinopathies. In addition, there are also a number of rare (<1%) immune-related adverse events that have also been reported following CPI treatment including Type I Diabetes and systemic lupus erythematosus (50, 185, 186). The causes and mechanisms of these various irAEs are an area of active investigation. We need to have predictive biomarkers for therapy response to maximize benefit as well as predictive biomarkers for irAEs to minimize risk, however, biomarkers for irAEs have been less vigorously investigated than biomarkers that predict therapy response. Nevertheless, as more and more immunotherapy are being deployed, some predictive immune signals are beginning to surface. For example, increased overall white blood cell count and eosinophil count with decreased relative lymphocyte count have been associated with higher grade irAEs (187, 188). Increased T-cell repertoire diversity is associated with more irAEs in patients treated with ipilimumab (189, 190). A post combination anti-CTLA-4 and anti-PD-1 therapy reduction in total peripheral B cells with an enrichment of CD21^lo^ PD-1^+^ memory B cells correlated with irAE development (191). Increases in circulating autoantibodies against self-antigens and mRNA gene expression signatures of immune activation have been correlated with impending irAEs (192–195). Finally, as mentioned, IL-17 levels may be associated with gastrointestinal toxicities (135). As most studies are small and not repeated in large-scale clinical trials, more work is needed before effective predictors of irAEs are clinically available.

## Novel Technologies and Assays Under Investigation

Biomarker assays that require the smallest amounts of accessible samples, for instance, blood and other bodily fluids from patient clinical samples, will inherently be less invasive and more likely to be implemented in a clinical setting. Blood biomarkers such as circulating tumor DNA may allow for more meaningfully analysis of patients tumor response with smaller blood volumes in comparison to approaches capturing circulating tumor cells and have been recently reviewed (196, 197). These approaches have the potential to overcome the tumor heterogeneity and sample quantity procurement limitations of tumor biopsies. A number of biotech companies including Grail, Freenome, and Guardant Health are utilizing this approach to create assay platforms that improve early cancer detection and immunotherapy responses for better outcomes. In addition, recent advancements in imaging are allowing for non-invasive evaluation of the tumor immune-microenvironment and have been recently well-reviewed (198). Using PET scan in conjunction with antibodies or antibody fragments labeled with PET-based radionuclides, scanners have the potential to detect T-cell subsets and effector molecules within tumors or lymphoid tissues and non-invasively monitor changes within the tumor microenvironment throughout the immunotherapy treatment process. Technologies such as this may help clinicians to better distinguish patients with true disease progression, which requires the timely transition to alternative treatments, from pseudoprogression, a phenomenon characterized by the transient increase in tumor size followed by a decrease, where immunotherapy should be continued.

## Discussion

Immunotherapies such as CPIs are demonstrating unprecedented response rates across all major types of metastatic skin cancers. Unlike traditional cancer treatment modalities, the durability of response seen in CPIs enabled clinician and patients alike to consider the possibility of cures in a historically highly resistant and fatal group of cancers. Building upon that success, efforts are underway to further improve response rates and more precisely deliver treatment to patients who are most likely to respond while monitoring treatment outcomes in a timely fashion. These efforts will likely serve to ameliorate the problem of high cost and unpredictability of cancer immunotherapy. This review attempts to summarize the tools currently available for clinical practice as well as technologies in emerging areas of discovery. As combination immunotherapy and associated biomarkers gain sophistication, efforts to prioritize the tools available and to standardize practice methods will require dedicated large studies. It is established that immune impairment is common among cancer patients, with the entity of metastatic skin cancer every so close to finally achieving improved clinical outcomes for its patients using immunotherapy, the mechanistic understanding of the tumor microenvironment and lessons learned from improving this field will hopefully benefit other disease entities as well.

## Author Contributions

JBr and JL contributed to the writing, figure design, and literature review. AD, JW, and JBl provided expert opinion and critical revision of the review. All authors contributed to manuscript revision, read and approved the submitted version.

### Conflict of Interest Statement

JB is CEO and President of Parker Institute for Cancer Immunotherapy; reports receiving commercial research funding from Juno/Celgene; has ownership interest in Celsius Therapeutics, Rheos Medicines, Arcus Biosciences, Solid Biosciences, Vir Biotechnology, and Quentis Therapeutics; and is a consultant/advisory board member for Quentis Therapeutics, Vir Biotechnology, Solid Biosciences, Arcus Biosciences, Rheos Medicines, Celsius Therapeutics, Pfizer, and Merck. The remaining authors declare that the research was conducted in the absence of any commercial or financial relationships that could be construed as a potential conflict of interest.
